# Magnetically responsive nanoplatform targeting circRNA circ_0058051 inhibits hepatocellular carcinoma progression

**DOI:** 10.1007/s13346-022-01237-z

**Published:** 2022-09-16

**Authors:** Song You, Zijin Luo, Niangmei Cheng, Ming Wu, Yongping Lai, Fei Wang, Xiaoyuan Zheng, Yingchao Wang, Xiaolong Liu, Jingfeng Liu, Bixing Zhao

**Affiliations:** 1 Department of Hepatobiliary Surgery, Fujian Medical University Cancer Hospital, Fujian Cancer Hospital, Fuzhou, 350014 People’s Republic of China; 2 The United Innovation of Mengchao Hepatobiliary Technology Key Laboratory of Fujian Province, Mengchao Hepatobiliary Hospital of Fujian Medical University, Fuzhou, 350025 People’s Republic of China; 3 Mengchao Med-X Center, Fuzhou University, Fuzhou, 350116 People’s Republic of China; 4 The Third Clinical Medical College, Fujian Medical University, Fuzhou, 350122 People’s Republic of China

**Keywords:** HCC, circ_0058051, siRNA, SPIONs

## Abstract

Circular RNAs (circRNAs) are a class of highly stable and closed-loop noncoding RNA that are involved in the occurrence and development of hepatocellular carcinoma (HCC). However, little is known about the therapeutic role of circRNAs in HCC. We found that high circ_0058051 expression was negatively correlated with the prognosis of HCC patients. Circ_0058051 knockdown attenuated the proliferation and colony formation, meanwhile inhibited migration of HCC cells. Circ_0058051 may be used as a target for HCC gene therapy. We synthesized a novel small interfering RNA (siRNA) delivery system, PEG-PCL-PEI-C14-SPIONs (PPPCSs), based on superparamagnetic iron oxide nanoparticles (SPIONs). PPPCSs protected the siRNA of circ_0058051 from degradation in serum and effectively delivered siRNA into SMMC-7721 cells. Meanwhile, intravenous injection of the PPPCSs/siRNA complex could inhibit tumor growth in the subcutaneous tumor model. In addition, the nanocomposite is not toxic to the organs of nude mice. The above results show that PPPCSs/si-circ_0058051 complex may provide a novel and promising method of HCC treatment.

**Graphical abstract:**

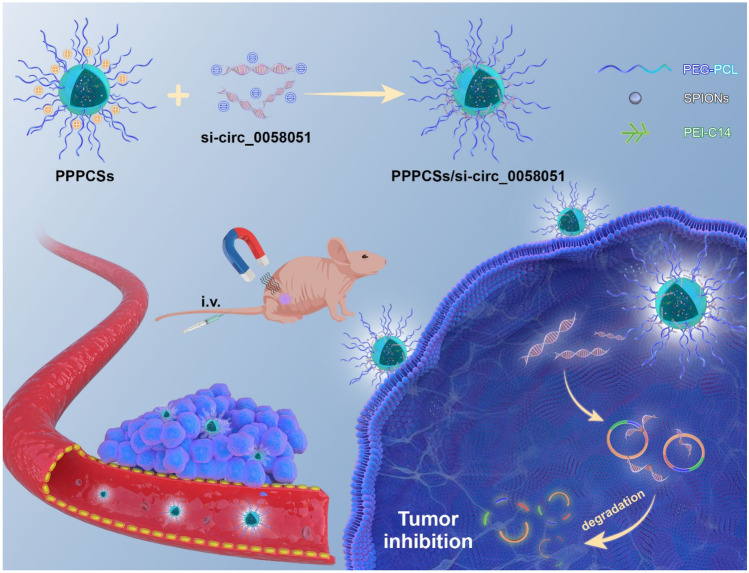

## Introduction

HCC ranks sixth and third in cancer-related incidence and mortality worldwide, respectively [1]. Although significant progress has been made in the treatment of HCC, including surgery, chemotherapy, radiotherapy, transarterial chemoembolization, and immune therapy, the overall 5-year survival rate of patients remains low, especially for patients with advanced stages [2]. Thus, developing novel and efficacious therapies will be of great significance to this malignant disease. Gene-targeted therapy has provided a promising new direction for its treatment.

Circular RNAs (circRNAs), a class of single-stranded covalent closed RNAs, are generated by back-splicing derived from precursor mRNA (pre-mRNA). The known (circRNAs) mainly consist of exons of protein-coding genes without 5′ end caps and 3′end poly(A) tails [3]. Growing studies have shown that circRNAs regulate the occurrence and progression of HCC by participating in physiological and pathological processes [4–6]. For example, up-regulation of hsa_circ_0003998 in HCC was significantly associated with malignant biological behavior, and sponge miR-143-3p promotes epithelial to mesenchymal transition of tumor [7]. In many cases, the expression levels of circRNAs are more abundant and stable than that of linear RNA [8]. Thus, circRNAs can be used as potential and promising targets for gene therapy.

Small interfering RNA (siRNA) is a double-stranded RNA, which can lead to efficient and specific gene silencing [9]. In the past few decades, siRNA has shown great potential in the treatment of human diseases, including cardiovascular disease, autoimmune disease, and cancers [10–12]. In August 2018, the world’s first siRNA drug (patisiran) was approved by the FDA for the treatment of patients with polyneuropathy caused by hereditary TTR-mediated amyloidosis, which indicates that siRNA-based gene therapy is shifting from research to clinical utility [13]. Currently, dozens of siRNA therapeutic drugs are undergoing clinical trials [14, 15]. Recently, ciRS-7 has been used as siRNA therapeutic targets in renal cell carcinoma, which achieved good results [16]. Therefore, using circRNA as a target for siRNA-based gene therapy has potential clinical value.

The poor pharmacokinetics and metabolic stability of RNA in vivo obviously limit the clinical application of RNA interference (RNAi) technology [17, 18]. Efficacious and safe siRNA delivery systems are the key issue for sequence-specific gene silencing [19]. Recently, superparamagnetic iron oxide nanoparticles (SPIONs) have been widely used in biomedicine due to their low toxicity and high magnetic stability [20, 21]. The main advantage of SPIONs is to increase the local drug concentration of the target through an exterior magnetic field (MF), which significantly improves the therapeutic effect, overcoming the technical problems in traditional treatment [22, 23]. However, upon cell entry, SPIONs generate oxidative stress and hydroxyl radicals, which limit their application in clinical trials [24]. Hence, many studies proposed a modification with polyethylene glycol-polycaprolactone (PEG-PCL) to prevent SPIONs from being cleared by macrophages, which increases the circulation time of SPIONs in the blood [25, 26]. Due to the polyanionic nature of siRNA, we synthesized polyethyleneimine derivative (PEI-C14) as a polycationic partner to condense siRNA, which was further co-assembled with PEG-PCL and SPIONs to fabricate the final hybrid delivery platform [27].

In this study, we first demonstrated that hsa_circ_0058051 (circ_0058051) could act as an oncogene that increases the proliferation and migration of HCC cells. Therefore, circ_0058051 might represent a potential target for HCC therapy. Meanwhile, we developed a magnetic nanoparticle-mediated delivery system with high degrees of stability and safety, which can effectively deliver circ_0058051 siRNA under an exterior MF to silence circ_0058051 in HCC. The results showed that PEG-PCL-PEI-C14-SPIONs (PPPCSs) effectively protect si-circ_0058051 from degradation by enzymes in serum and tissues. Meanwhile, the PPPCSs/si-circ_0058051 complex significantly inhibited tumorigenesis and progression of HCC by silencing circ_0058051 in vitro and in vivo.

## Result

### Characterization and expression analysis of circ_0058051 in HCC

**Fig. 1:**
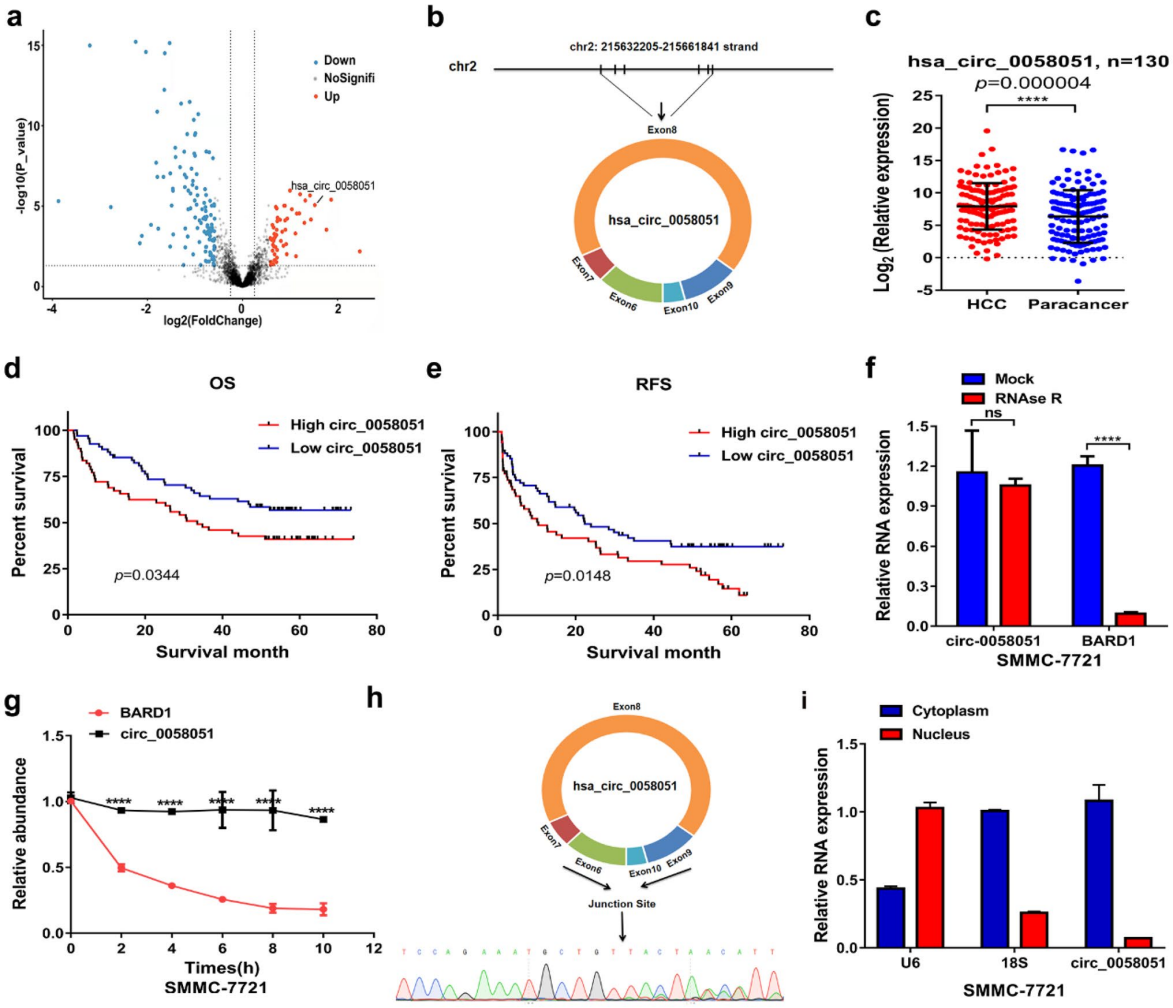
The identification of circ_0058051 in HCC. **a** Volcano plot of the differences in circRNA abundance between HCC tumor tissues and matched peritumor tissues. **b** The genomic loci of circ_0050051. **c** The level of circ_0058051 in HCC tissues and adjacent tissues was detected by qRT-PCR (****P < 0.0001). **d** Kaplan–Meier overall survival analysis of HCC patients with high circ_0058051 level and low circ_0058051 level. **e** Kaplan–Meier recurrence-free survival analysis of HCC patients with high circ_0058051 level and low circ_0058051 level. **f** The level of circ_0058051 and BARD1 in SMMC-7721 cells treated with RNase R (****P < 0.0001). **g** The level of circ_0058051 and BARD1 in SMMC-7721 cells treated with actinomycin D at different time points (****P < 0.0001). **h** Sanger sequencing was performed to determine junction site. **i** The localization of circ_0058051 in SMMC-7721 cells were determined by nuclear and cytoplasmic separation assay

To characterize the circRNA profile involved in HCC progression, whole transcriptome sequencing was performed on 61 pairs of HCC tumors and matched peritumor tissues. A total of 173 circRNAs were differentially expressed in HCC, including 55 up-regulated circRNAs and 118 down-regulated circRNAs (Fig. 1a). Among them, circ_0058051 was significantly up-regulated in HCC tissues. CircBase revealed that circ_0058051 was generated from exons 6–10 of the BARD1 gene (Fig. 1b). We then validated the expression of circ_0058051 in HCC by RT-qPCR. As expected, the expression of circ_0058051 was significantly up-regulated in HCC tissues as compared with adjacent cancers (Fig. 1c). Further clinical pathology data analysis shows that higher circ_0058051 expression was significantly correlated with vascular invasion (*P* = 0.030), tumor differentiation (*P* = 0.004), and TNM stage (*P* = 0.009) (Table 1). In addition, Kaplan–Meier survival analysis revealed that the expression of circ_0058051 was negatively correlated with the prognosis of HCC patients (Fig. 1d, e). To confirm the existence of circ_0058051, SMMC-7721 cells were treated with RNase R exonuclease and actinomycin D, respectively. The results of qRT-PCR showed that circ_0058051 was more stable and resistant than linear BARD1 (Fig. 1f, g). Moreover, the junction site was further validated by sanger sequencing (Fig. 1h). Cytoplasmic separation assay showed that circ_0058051 was mainly located in the cytoplasm (Fig. 1i). Taken together, our results showed that circ_0058051 was highly expressed in HCC tissues and was an abundant and stable circRNA expressed in HCC cells.

**Table 1:** circ-0058051 abnormal expression correlates with clinicopathological factors of HCC patients

**Clinicopathological indexes**	**circ-0058051 expression**		***X***^2^	***P***
	**Low (*n* = 69)**	**High (*n* = 61)**		
Age (year)				
≤ 50	31	34	1.513	0.219
> 50	38	27		
Gender				
Male	59	53	0.052	0.820
Female	10	8		
Tumor size				
≤ 5 cm	38	29	0.735	0.391
> 5 cm	31	32		
Tumor number				
Single	60	45	3.642	0.057
Multiple	9	16		
Vascular invasion				
No	38	22	4.706	0.030*
Yes	31	39		
Differentiation				
I–II	24	8	8.192	0.004*
III–IV	45	53		
Liver cirrhosis status				
No	21	10	3.515	0.061
Yes	48	51		
Serum HBV level (cps/ml)				
≤ 500	14	8	1.179	0.278
> 500	54	52		
TNM stage				
I–II	62	44	6.757	0.009*
III–IV	7	17		

### Circ_0058051 silencing inhibits the malignant biological behavior of HCC cells

**Fig. 2:**
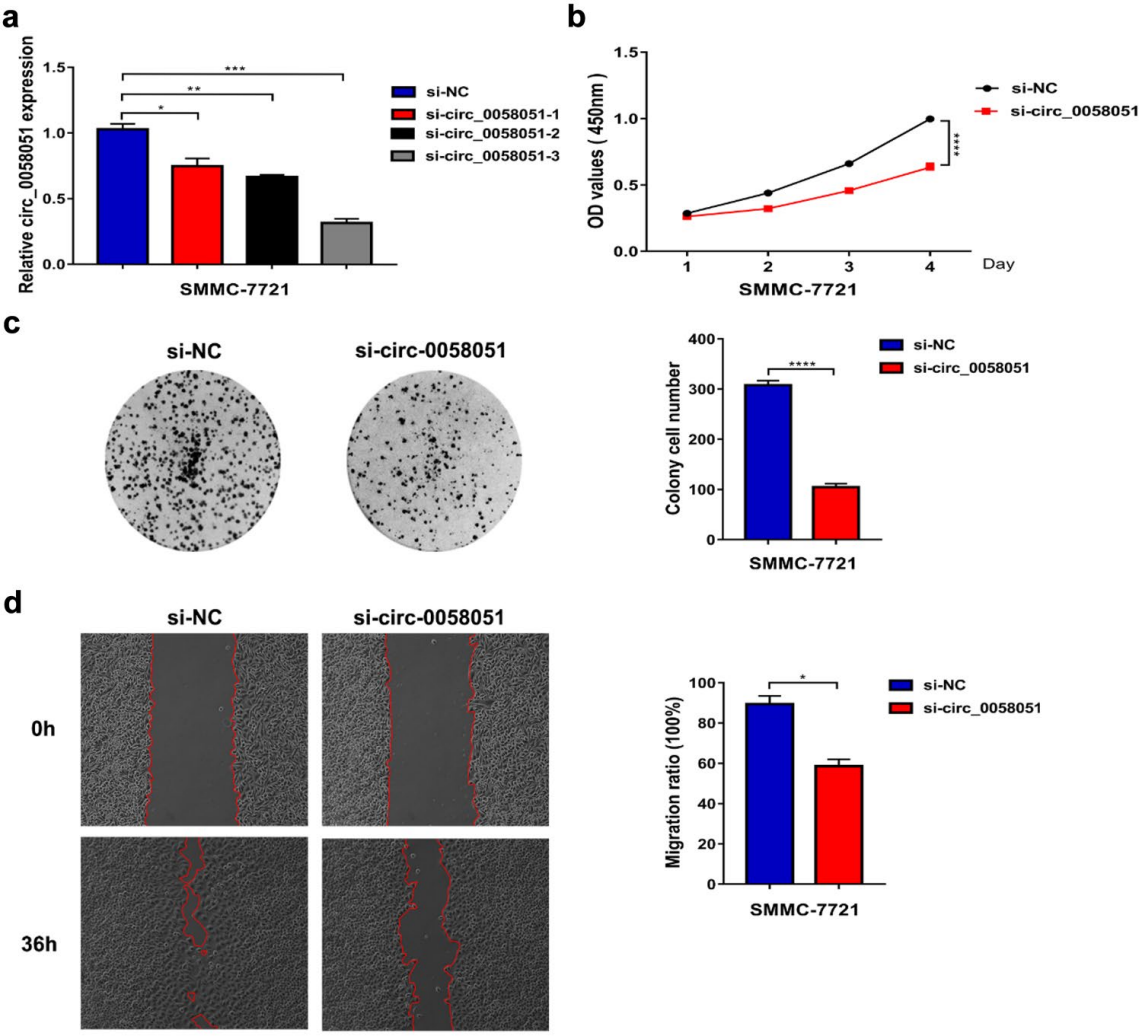
The function of circ_0058051 in HCC cells. **a** qRT-PCR detected the expression of circ_0058051 in circ_0058051 knockdown SMMC-7721 cells (***P < 0.001). After circ_0058051 silencing, cell proliferation was determined by **b** CCK8 and **c** colony formation assays. **d** Wound-healing assay was utilized to evaluate cell migration. (*P < 0.05; ****P < 0.0001)

To investigate the biological function of circ_0058051 in HCC, we designed and constructed three circ_0058051 siRNA sequences and used cell transfection assay to verify the knockdown effect (Fig. 2a). The si-circ_0058051-3 with the best knockdown effect was selected for subsequent experiments. Compared with the control group, circ_0058051 silencing significantly inhibited the proliferation, colony formation, and migration of HCC cells (Fig. 2b–d). Taken together, these results indicated that circ_0058051 silencing could inhibit the malignant biological behavior of HCC in vitro.

### Characterization of PPPCSs

**Fig. 3:**
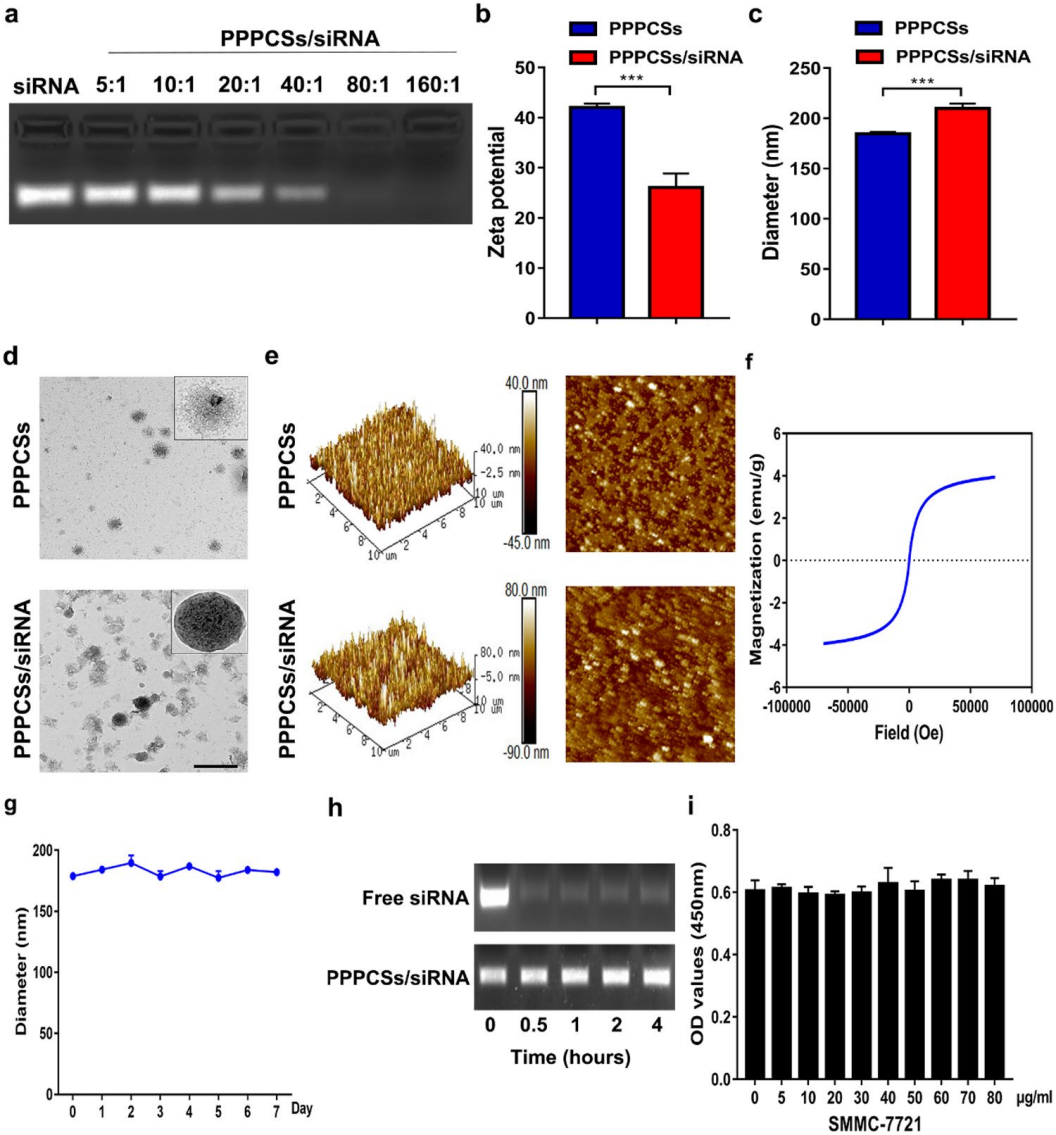
Characterization of PPPCSs/siRNA complex. **a** Gel retarding assay of PPPCSs/siRNA complex. **b** Surface charge of PPPCSs and PPPCSs/siRNA (***P < 0.001). **c** The diameter of PPPCSs and PPPCSs/siRNA in water. **d** TEM images of PPPCSs and PPPCSs/siRNA. **e** AFM images of PPPCSs and PPPCSs/siRNA. **f** Hysteresis loop of PPPCSs at 300 K. **g** Stability curve of PPPCSs. **h** Serum nucleases degradation assay. **i** Cytotoxicity of nanomaterials was assessed by CCK8 assay. Scale bar = 500 μm

Based on the new insights into tumor pathogenesis, gene targeting therapy has gained attention as a potential approach for cancer treatment. siRNA-loaded SPIONs, in combination with the external magnetic field (MF), that is, MF-mediated targeting, can increase drug accumulation in target tissues and reduce systemic toxicity. The siRNA loading capacity of PPPCSs was evaluated by agarose gel electrophoresis assay. When the mass ratio of siRNA to PPCSs was 1:80, no siRNA bands could be observed (Fig. 3a). Thus, this indicated ratio was used in subsequent experiments. As shown in Fig. 3b, zeta potential of PPPCSs decreased from + 42.07 ± 0.72 to + 26.03 ± 2.80 mV after loading siRNA. The core structure of the prepared PPPCSs was SPIONs and had a diameter of 185.27 ± 1.00 (Fig. 3c, d). After loading siRNA, the PPPCSs/siRNA diameter was 210.00 ± 4.73. In addition, we used Image J software to measure the diameter of PPPCSs (187.00 ± 10.44) and PPPCSs/siRNA (206.92 ± 2.78) in the TEM images to further verify this fact. The results of the atomic force microscope (AFM) showed that the height of PPPCSs/siRNA was significantly higher than that of PPPCSs (Fig. 3e). Hysteresis loop analysis revealed that PPPCSs exhibited superparamagnetic behavior with a magnetization saturation of 3.94 emu/g (Fig. 3f). Moreover, the diameter of PPPCSs did not differ significantly within 1 week in PBS containing 10% serum, indicating the high stability of PPPCSs (Fig. 3g). The protection of siRNA from degradation is the key challenge for delivering vectors. Hence, a serum enzyme degradation assay was performed. As shown in Fig. 3h, after incubated in DMEM with 20% serum for 4 h, the siRNA band extracted from the PPPCSs/siRNA complex still maintained the same brightness as the control group. These results showed that PPPCSs can be used as a delivery vector for siRNA and protect siRNA from degradation by serum enzymes. Cytotoxicity is the main obstacle to the use of nanomaterials. As shown in Fig. 3i, the proliferation ability of SMMC-7721 cells was not observed to decrease after treatment with PPPCSs, even at a concentration above 80 μg/ml for 24 h.

### Uptake of PPPCSs/siRNA in HCC cells

**Fig. 4:**
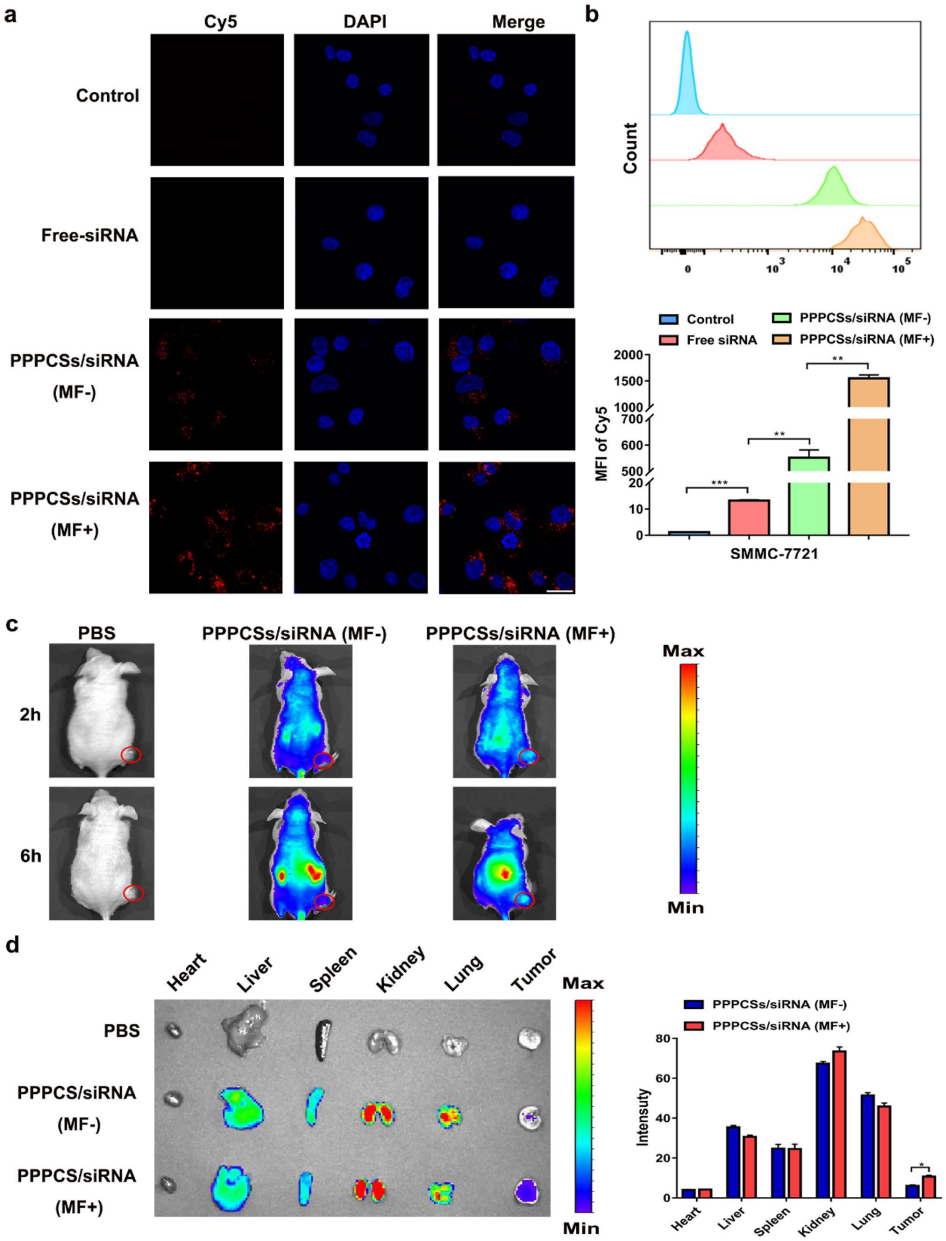
Cellular uptake and biodistribution of PPPCSs/siRNA complex. **a** siRNA-Cy5 uptake in different treatment groups. **b** Flow cytometry analysis of siRNA-Cy5 uptake in different treatment groups (**P < 0.01; ***P < 0.001). **c** In vivo imaging of nude mouse HCC subcutaneous tumor model at different time points after tail vein injection of PBS, PPPCSs/siRNA-Cy5 (MF −) and PPPCSs/siRNA-Cy5 (MF +). **d** Imaging of major organs and tumors isolated from nude mice tail vein injection of PBS, PPPCSs/siRNA-Cy5 (MF −), and PPPCSs/siRNA-Cy5 (MF +) (*P < 0.05). Scale bar = 20 μm

Next, we examined the internalization of the PPPCSs/siRNA complex in SMMC-7721 cells. The Cy5-labeled siRNA was loaded in the PPPCSs nanoplatforms to track their uptake in cells. As shown in Fig. 4a, under the exterior MF, the uptake of siRNA-Cy5 in the PPPCSs/siRNA (MF +) group was significantly higher than those of the negative control, free siRNA, and PPPCSs/siRNA (MF −) groups. Flow cytometry also confirmed that compared with the control group and free siRNA group, the intracellular uptake in the PPPCSs/siRNA groups was significantly increased (Fig. 4b). Additionally, the mean fluorescence intensity of Cy5 in the PPPCSs/siRNA (+ MF) group was significantly higher than that in the PPPCSs/siRNA (− MF) group (Fig. 4b). These results indicate that the PPPCSs/siRNA complex can be taken up into the cell more quickly and efficiently under the exterior MF.

### In vivo biodistribution via tail vein injection

To investigate the distribution of PPPCSs/siRNA complex in vivo, we injected PBS, PPPCSs/siRNA-Cy5 (MF −), and PPPCSs/siRNA-Cy5 (MF +) into nude mice through the tail vein. After 2 and 6 h of injection, the fluorescence image showed that the fluorescence intensity of the tumor site was significantly higher in the PPPCSs/siRNA (MF +) group than the PBS group and the PPPCSs/siRNA (MF −) group (Fig. 4c). After 24 h of injection, the isolated tumor image showed that the PPPCSs/siRNA (MF +) group exhibited a higher level of fluorescent intensity (Fig. 4d). The fluorescence intensity of siRNA-Cy5 in the tumor area confirmed that MF can increase the accumulation of PPPCSs/siRNA at the tumor site.

### PPPCSs/siRNA effectively inhibited the proliferation of HCC cells in vitro

**Fig. 5:**
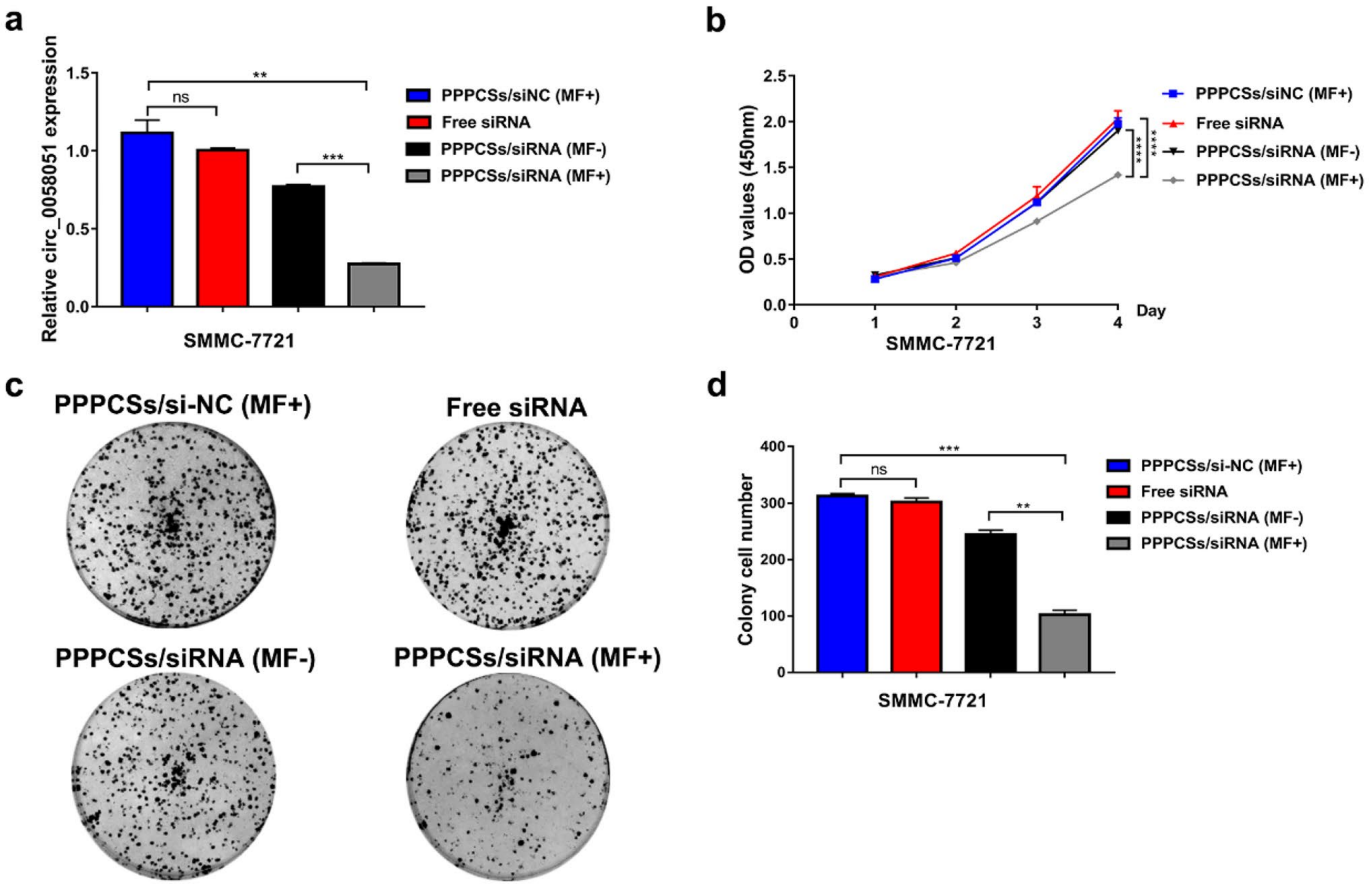
The proliferation of HCC cells was inhibited by PPPCSs/siRNA complex. **a** qRT-PCR analyzed circ_0058051 level after transfection. **b** Anti-proliferation effect of PPPCSs/siRNA was evaluated by CCK8 assay (****P < 0.0001). **c** Colony formation was performed to measure the effect of PPPCSs/siRNA complex. **d** Colony Number of each treatment group (**P < 0.01; ***P < 0.001)

CCK8 and colony formation assay were used to evaluate whether PPPCSs/siRNA has the ability to inhibit the proliferation of HCC cells. As shown in Fig. 5a, we treated SMMC-7721 cells with PPPCSs/siRNA complexes to significantly block the expression of circRNA_0058051. After PPPCSs/siRNA (MF +) treatment, the viability of SMMC-7721 cells was significantly reduced, indicating that silencing circRNA_0058051 inhibited the proliferation of SMMC-7721 cells (Fig. 5b). Colony formation assay showed that silencing circRNA_0058051 reduced the number of colonies of SMMC-7721 cells (Fig. 5c, d). These results indicate that PPPCSs/siRNA complex can suppress the progression of HCC in vitro.

### PPPCSs/siRNA effectively inhibited the proliferation of HCC in subcutaneous tumors

**Fig. 6:**
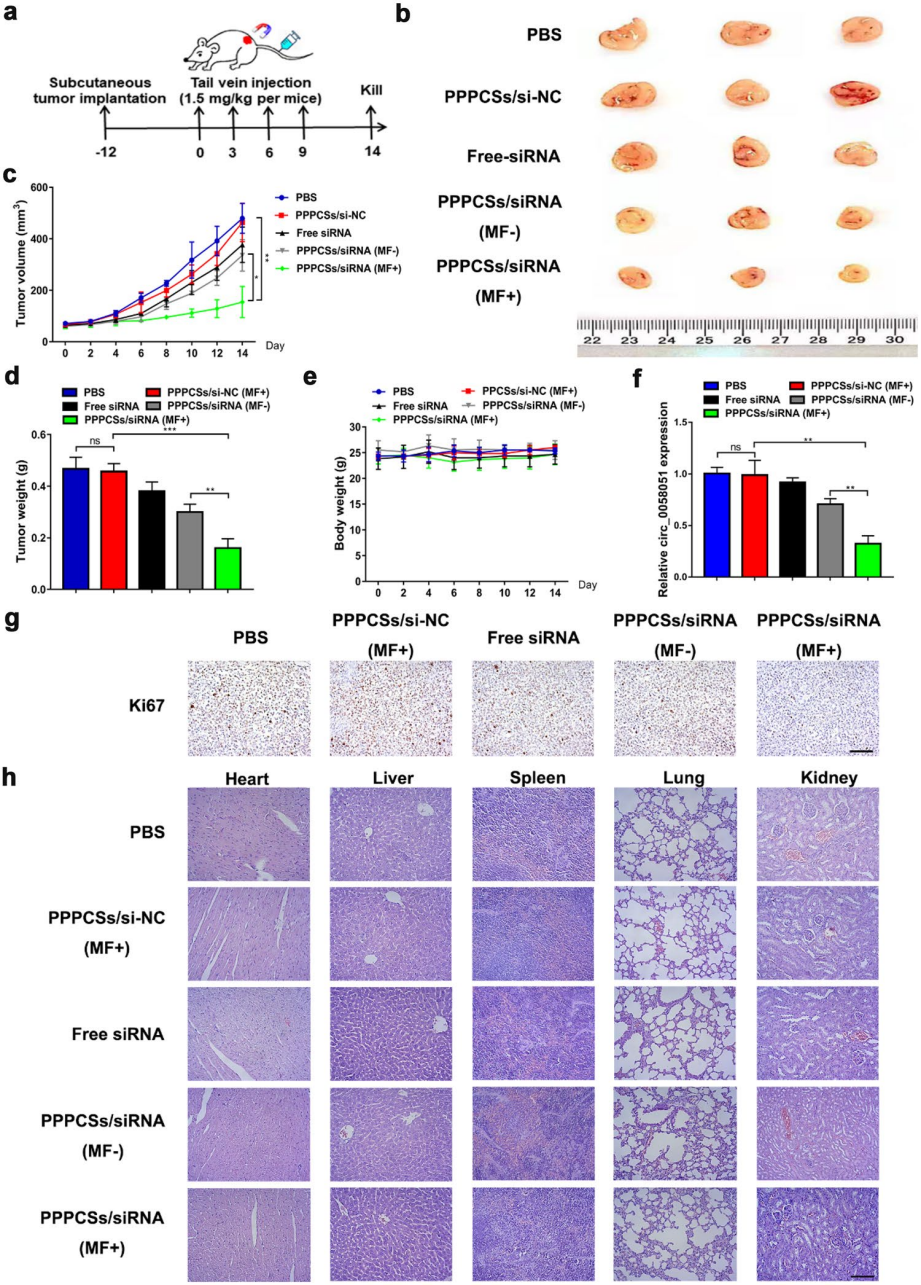
PPPCSs/siRNA complex inhibited HCC proliferation in vivo. **a** Schematic view of PPPCSs/siRNA treatment. **b, c** Tumor volume of each treatment group (*P < 0.05; **P < 0.01). **d** Tumor weight of each treatment group (**P < 0.01; ***P < 0.001). **e** Body weight of each treatment group. **f** The levels of circ_0058051 in PBS, PPPCSs/si-NC, free siRNA, PPPCSs/siRNA (MF −), or PPPCSs/siRNA (MF +) groups were detected by qRT-PCR. **g** Ki67 staining of tumor samples form PBS, PPPCSs/si-NC, free siRNA, PPPCSs/siRNA (MF −), or PPPCSs/siRNA (MF +) mice. Scale bar = 50 μm. **h** H&E staining of heart, liver, spleen, lung, and kidney in each group. Scale bar = 50 μm

We further investigated the effect of PPPCSs/si-circ_0058051 nanocomplexes on the in vivo HCC growth in the xenogeneic subcutaneous tumor model. Five groups of drugs were injected into mice through the tail vein on the 12, 15, 18, and 21 days after implantation (Fig. 6a). Compared with the other four groups, the tumor growth in the PPPCSs/siRNA (MF +) group was significantly inhibited (Fig. 6b, c). Meanwhile, the tumor weight showed that the PPPCSs/siRNA (MF +) group was significantly smaller than those of the PBS, PPPCSs/si-NC (MF +), free siRNA, and PPPCSs/siRNA (MF −) (Fig. 6d). As seen from Fig. 6e, no significant changes were observed in the body weight of nude mice during the treatment period, indicating that the PPPCSs/siRNA complex has no obvious toxicity in vivo. Next, the knockdown effect of PPPCSs/siRNA was verified by detecting the expression of circ_0058051 (Fig. 6f). Free siRNA did not inhibit the expression of circ_0058051. PPPCSs/siRNA (MF +) group more significantly inhibited the expression of circ_0058051 compared to PPPCSs/siRNA (MF −). In addition, IHC analysis showed that free siRNA treatment did not downregulate ki67 levels in tumor tissues (Fig. 6g). The expression of ki67 in PPPCSs/siRNA (MF +) group tumor tissues was significantly decreased compared to PPPCSs/si-NC (MF +) and PPPCSs/siRNA (MF-). Meanwhile, the toxicity of the complex in vivo was studied by H&E staining the main organs of nude mice. As shown in Fig. 6h, compared with PBS, negative control PPPCSs/si-NC and free siRNA group, no abnormalities were found in PPPCSs/siRNA (MF-) and PPPCSs/siRNA (MF +).

## Discussion

HCC is one of the cancers with a high fatality rate, which makes its treatment still facing a great challenge [28]. Recently, siRNA-based gene therapy has attracted increasing attention [29]. However, its clinical application is limited by the lack of effective therapeutic targets and powerful siRNA delivery vectors [30]. In this study, we first found that circ_0058051, as a new oncogenic factor, can participate in HCC progression. Next, we developed a non-toxic and efficient magnetic targeted delivery vector called PPPCSs. By delivering siRNA to target silencing circ_0058051, the progression of HCC was significantly inhibited in vivo and in vitro.

Recently, many studies have shown that circRNAs can regulate the proliferation, angiogenesis, distant metastasis, and drug resistance of HCC, which makes circRNAs useful as diagnostic markers and therapeutic targets [31–33]. So far, there has been no relevant report on circRNA-based gene therapy on cancer due to the lack of an efficacious target. In this study, our results demonstrated that circ_0058051 was markedly up-regulated in HCC tissues, and circ_0058051 silencing reduced the proliferation and migration of SMMC-7721 cells in vitro. Thus, circ_0058051 might be used as a valuable therapeutic target against the progression of HCC.

Small interfering RNA is the most promising treatment in the field of gene therapy [34]. Today, three siRNA drugs, patisiran, givosiran, and inclisiran, have been approved by the FDA for the treatment of peripheral nerve diseases, acute hepatic porphyria, and hyperlipidemia, respectively [35, 36]. The safety and effectiveness of delivery vectors are critical to the development of siRNA drugs [37]. For the delivery of siRNA in vivo, various nonviral nanoparticle carrier systems have been explored and developed, including polymers, inorganic nanomaterials, exosome-mimetic nanovesicles, and liposomes [38–40]. For example, Liu et al. reported using natural halloysite nanotube to deliver receptor-interacting protein kinase 4 siRNA for bladder cancer therapy [41]. In this research, we constructed a novel and safe siRNA delivery system based on SPIONs, which can protect siRNA from degradation by serum enzymes and achieve targeted delivery of siRNA, thus further applied to systemic intravenous administration.

Under the exterior MF, PPPCSs could efficiently and safely load si-circ_0058051 to reach HCC tissue and achieve specific gene silencing. PPPCSs were degraded in vivo to release iron ions. Serum-free iron was metabolized by the liver and then excreted in the urine [42]. The effect of free iron on tissue oxidative stress needs to be considered for the safe application of nanoparticles [43]. Jain et al. reported that injected iron induced a transient oxidative stress response in tissues and then returned to normal [42]. Our pathological data also indicated that PPPCSs/si-circ_0058051 did not cause off-target toxicity and tissue damage. In conclusion, our work has not only discovered a new therapeutic target but also developed a targeted therapeutic siRNA nanocomposite, which will provide great potential for the clinical treatment of HCC.

## Conclusion

The magnetically responsive nanoplatform targeting circRNA circ_0058051 can inhibit HCC progression both in vitro and in vivo. Our study may represent a promising gene therapeutic strategy for HCC.

## Materials and method

### HCC tissue

Human HCC tissues were collected from patients who underwent HCC resection at Mengchao Hepatobiliary Hospital of Fujian Medical University from 2016–2018. The research protocol obtained the informed consent of all patients and was approved by the ethics committee of Mengchao Hepatobiliary Hospital of Fujian Medical University (No.2021_068_01).

### CircRNA sequencing

Total RNA was extracted from 61 pairs of samples (61 HCC and matched peritumor tissues) using a kit (TransGen Biotech, China). CircRNA sequencing was performed after the removal of ribosomal RNA and linear RNA. Transcriptome sequencing results were analyzed using CIRI software. Differentially expressed circRNAs were screened following the criteria of fold change > 1.5 and *P* < 0.05.

### Cell line and cell culture

Human HCC SMMC-7721 cells were cultured in Dulbecco’s modified Eagle’s medium (DMEM) (Gibco, USA) supplemented with 10% fetal bovine serum (FBS) (Invitrogen, USA), 100 IU/ml penicillin and streptomycin (Gibco, USA).

### Material synthesis and characterization

1,2-Epoxytetradecane and PEI25000 were dissolved in absolute ethanol at a mass ratio of 2:1 and reacted at 90 °C for 48 h. The mixture was cooled to room temperature and purified to obtain PEI-C14. The SPIONs were extracted with absolute ethanol. Iron acetylacetonate (2 mM), oleic acid (6 mM), and oleylamine (4 mM) were added to 20 ml of phenyl ether and stirred thoroughly in a nitrogen atmosphere. Next, cetyl alcohol (10 mM) was added and heated to 210 °C. The mixture was refluxed for 2 h and then cooled to room temperature. The SPIONs were extracted with absolute ethanol. After centrifugation at 7000 rpm for 10 min, SPIONs were collected and dissolved in n-hexane. SPIONs (1 mg), PEG-PCL (10 mg), and PEI-C14 (1 mg) were added to 1 ml of dichloromethane. Next, 5 ml of ultrapure water was added to the mixture. Finally, the dichloromethane is removed by rotary evaporation. PPPCSs/siRNA complex was collected and stored at 4 °C. The diameter of PPPCSs and PPPCSs/siRNA was observed with dynamic light scattering and transmission electron microscope (TEM). An atomic force microscope (AFM) was used to perform the AFM-based characterization of PPPCS and PPPCS/siRNA. SQUID magnetometer (Quantum Design, USA) was utilized to measure the magnetic properties of PPPCSs. The change in diameter of PPPCSs in PBS containing 10% FBS was measured daily for one week to determine the stability of PPPCSs.

### Screening of siRNA sequences

Three circ-0058051 siRNA duplexes and a negative control (si-NC) (Table 2) were synthesized by SunYa (Fuzhou, China) and transfected into SMMC-772a1 cells with lipofectamine 3000. After transfection, the expression levels of circ-0058051 in SMMC-7721 cells were analyzed by RT–PCR. The most effective sequence of circ-0058051 siRNA was selected for further experiments.circ-0058051 abnormal expression correlates with clinicopathological factors of HCC patients

**Table 2:** siRNA sequence list

si-circ_0058051-1 sense	GCUGUUACUAACAUUCUGAGATT
si-circ_0058051-1 antisense	UCUCAGAAUGUUAGUAACAGCTT
si-circ_0058051-2 sense	GAAAUGCUGUUACUAACAUUCTT
si-circ_0058051-2 antisense	GAAUGUUAGUAACAGCAUUUCTT
si-circ_0058051-3 sense	CUCCAGAAAUGCUGUUACUAATT
si-circ_0058051-3 antisense	UUAGUAACAGCAUUUCUGGAGTT

### Preparation of PPPCSs/siRNA complex

The negatively charged siRNA was mixed with PPPCSs in ultrapure water and left at room temperature for 30 min. The mass ratio of PPPCSs to siRNA was 1:1, 5:1, 10:1, 20:1, 40:1, 80:1, and 160:1. Agarose gel electrophoresis (2%) is used to verify the most suitable concentration of the PPPCSs/siRNA complex.

### siRNA degradation assay

For the siRNA degradation assay, free siRNA or PPPCSs/siRNA was placed in DMEM containing 20% FBS at 37 °C for 0 h, 0.5 h, 1 h, 2 h, and 4 h. Each sample was prepared with 10 × DNA loading buffer with SDS (Takara, Japan) and placed in 2% nucleic acid gel wells and electrophoresed at 120 mV for 15 min. The resulting gel was observed and analyzed by the gel imaging analysis system.

### RNA extraction and quantitative real-time PCR (qRT-PCR)

Total RNA from SMMC-7721cells and HCC samples were extracted with TransZol Up Plus RNA Kit (Trans, China) according to the manufacturer’s instructions. cDNA was synthesized using the Hifair® II 1st Strand cDNA Synthesis Kit (YESEN, China) according to the manufacturer’s instructions. The expression values of circ_0058051 and 18S rRNA were detected by using SYBR Green qPCR Mix (DBI, Germany) in a StepOnePlus™ Real-Time PCR System (ABI, USA). 18S rRNA was used as an internal reference. The primers for circ_0058051 and 18S rRNA were as follows: circ_0058051: 5′-ACACCACCGGGTATCAAA-3′ (forward); 5′-GCTCACATCCTCCTAAACA-3′ (reverse); 18S rRNA: 5′-AGAAACGGCTACCACATCCA-3′ (forward); 5′-CACCAGACTTGCCCTCCA-3′ (reverse).

### Cell cytotoxicity assay

SMMC-7721 cells were seeded in 96-well plates at a density of 5000 cells per well for cytotoxicity experiments. PPPCSs (5, 10, 20, 30, 40, 50, 60, 70, and 80 μg/ml) conjugated with 50 nM siRNA was used to treat SMMC-7721 cells for 24 h. Next, washing cells were with PBS and the number of living cells was tested by using CCK8 according to the manufacturer’s instructions. The untreated group was used as a negative control.

### Cell transfection assay

For the cell transfection assay, the transfection solubility of siRNA was 50 nM. PPPCSs and siRNA were mixed in ultrapure water for 30 min at room temperature, using the weight ratio of PPPCSs to siRNA was 1:80. HCC cells were incubated with the PPPCSs/siRNA complex for 4 h. After 24 h, PPPSCs/siRNA was incubated again for 4 h.

### Cellular uptake

For the cellular uptake assay, a fluorescent dye Cy5, was attached to the sense strand of circ_0058051 siRNA at the 5′ end. A total of 15 × 1,047,721 cells were seeded in a confocal dish. After 24 h, the SMMC-7721 cells were treated with free siRNA-Cy5, PPPCSs/siRNA-Cy5 (MF −), and PPPCSs/siRNA-Cy5 (MF +) for 4 h. Then the above four groups of SMMC-7721 cells were fixed with 4% paraformaldehyde and incubated in 4,6-diamidino-2-phenylindole (1:1000) for 10 min. Following PBS washes, the confocal dishes were observed and photographed under the laser scanning confocal microscope (Zeiss, Germany).

### Flow-cytometry assay

Approximately, 5 × 10^5^ SMMC-7721 cells were placed in 6-well plate and cultured at 37 °C for 24 h. Then cells were incubated with free siRNA-Cy5, PPPCSs/siRNA (MF −), and PPPCSs/siRNA (MF +) for 4 h. The fluorescence intensity of Cy5 in each group was detected by flow cytometry, respectively. Each experiment was repeated three times independently. The results were analyzed in the FlowJo_V10 software.

### Cell proliferation and colony formation assay

After cell transfection, SMMC-7721 cells (2 × 10^3^/well) were seeded into 96-well plates and cultured with 100 μl of complete medium. The absorbance values were detected by using Cell Counting Kit-8 (CCK8) in a microplate reader (Molecular Devices). For colony formation, 1000 cells were added to each well of the six-well plate. Two weeks later, colonies were fixed in 4% paraformaldehyde for 10 min and stained with crystal violet.

### Magnetic targeting

For magnetic targeting in vitro, cells were treated with PPPCSs/siRNA with a 4000 gs magnet attached to the bottom of the dish. For magnetic targeting in vivo, a 1 cm diameter disc neodymium magnet (4000 gs) was gently placed on the surface of the tumor and secured using autoclave tape. After 12 h of nanoparticle injection, the neodymium magnets were removed.

### In vivo fluorescence imaging

The PPPCSs/siRNA (1.5 mg/kg siRNA per mice) complex was injected into nude mice through the tail vein. The distribution of the complex at different time points (2 and 6 h) in the body was observed through the intravital imaging system. After 24 h, the mouse was euthanized, and the main organs (heart, liver, spleen, lung, and kidney) and tumor were removed and imaged under the same system.

### Animal study

Four-week-old male nude mice were used in animal therapy models. A total of 5 × 10^6^ SMMC-7721 cells in 66 μl of basal medium containing 33 μl of matrigel were injected under the skin of the back of the nude mouse. When the tumor volume reached 70–80 mm^3^, nude mice were randomly divided into the PBS, PPPCSs/si-NC, free siRNA, PPPCSs/siRNA (MF −), and PPPCS/siRNA (MF +) groups (*n* = 3). Next, different siRNA formulations (1.5 mg/kg siRNA per mice) or same quality PBS (30 μg per mice equivalent) was injected through the tail vein once every three days for a total of four times. The nude mice were sacrificed, and the differences between the groups were counted.

### Hematoxylin and eosin (H&E) staining and immunohistochemistry (IHC)

H&E staining and IHC were performed as previously described [44]. Antibody Ki-67 (1:200, ab16667, Abcam) was used for IHC detection.

### Statistical analysis

All data were represented by mean ± SD and analyzed by the GraphPad Prism software. A Student *T*-test was used for comparison between the two groups. The Kaplan–Meier method was used to calculate the survival rate. *P* < 0.05 was considered to be statistically significant.

## Data Availability

Any data or material that support the findings of this study can be made available by the corresponding author upon request.

## Materials availability

Any data or material that support the findings of this study can be made available by the corresponding author upon request.
